# Endotoxin tolerance enhances breast cancer aggressiveness and alters inflammatory marker expression in tumor and spleen of mice

**DOI:** 10.3389/fimmu.2026.1660646

**Published:** 2026-03-19

**Authors:** Konkonika Roy, Bartosz Maciejewski, Tomasz Jędrzejewski, Paulina Spisz, Justyna Sobocińska, Melania Di Pentima, Benedetta Passeri, Sylwia Wrotek

**Affiliations:** 1 Department of Immunology, Faculty of Biology and Veterinary Sciences, Nicolaus Copernicus University, Torun, Poland; 2 Pathology Unit, Department of Veterinary Science, University of Parma, Parma, Italy

**Keywords:** cancer, endotoxin tolerance, immunosuppression, inflammation, tumor microenvironment

## Abstract

Endotoxin tolerance (ET) is an immunological state in which repeated exposure to endotoxins such as lipopolysaccharide (LPS) leads to reprogramming of the immune system and a diminished inflammatory response. In this study, we used a murine model to explore the role of ET in breast cancer progression, hypothesizing that ET may foster a tumor-permissive immune environment. We compared endotoxin tolerant breast cancer-bearing mice (ETBC group) with non-endotoxin tolerant breast cancer-bearing controls (BC group). ETBC mice exhibit significantly faster tumor progression and earlier disease onset. Hematological analysis revealed reduced leukocyte counts in the ETBC group, indicating compromised immune cell recruitment. Additionally, ETBC mice showed decreased spleen weight relative to that in the BC group, further supporting systemic immune suppression. Gene expression profiling in both spleen and tumor tissues revealed marked immunological alterations in ETBC mice. In the spleen, there was notable downregulation of key pro-inflammatory cytokines, including interleukin (IL) 6 and interferon (IFN) γ. Conversely, genes associated with immune modulation and tumor progression such as IL-1β, inducible nitric oxide synthase (NOS2), cyclooxygenase (COX) 2, vascular endothelial growth factor (VEGF), and colony stimulating factor 1 (CSF-1) were upregulated. Notably, IL-1β, NOS2, COX-2, IL-10, and VEGF were consistently upregulated in tumor tissues of ETBC mice. We conclude that ET not only impairs immune surveillance but also reshapes the tumor microenvironment in favor of cancer growth. This highlights the potential role of ET in oncology and suggests that its modulation could represent a novel avenue for therapeutic intervention.

## 1 Introduction

Endotoxin tolerance (ET) is a phenomenon in which prior exposure to endotoxins, such as lipopolysaccharide (LPS), reprograms macrophages and alters their response to subsequent endotoxin challenges (1, 2). When primed with endotoxins, macrophages exhibit heightened reactivity and produce elevated levels of inflammatory mediators (3, 4). In contrast, endotoxin tolerant macrophages demonstrate a diminished response, characterized by the reduced production of these mediators. Changes in inflammatory markers also lead to systemic alterations across the entire organism, with the most noticeable being the weakening or absence of fever, as evidenced by attenuated body temperature changes following the administration of a pyrogenic dose of endotoxin (5–7). Fever itself plays a complex role in immune regulation, directly modulating immune responses by enhancing the activity of cytotoxic T cells and natural killer cells (8, 9). However, the inability to trigger fever due to ET may not only impair immune function but also obscure important warning signals of immune dysregulation, potentially affecting the body’s capacity to respond to tumor development (10–12).

Breast cancer is one of the most prevalent and deadly cancers worldwide, affecting millions of people annually. Despite advances in detection and treatment, breast cancer remains a leading cause of cancer-related deaths, primarily because of its potential for metastasis and resistance to therapies (13, 14). Understanding the factors that drive breast cancer progression, particularly those linked to the immune system, is essential for developing innovative therapeutic strategies. The immune system plays a dual role in cancer by promoting the elimination of tumor cells and paradoxically creating a microenvironment that supports tumor growth and metastasis (15–18). Given the complex interplay between inflammation and cancer, exploring how ET-related immune reprogramming influences tumor development may offer valuable insights for advancing therapeutic strategies.

To date, most studies exploring the impact of ET on cancer progression have been limited to *in vitro* models (10) that fail to capture the full complexity of interactions within a living organism. To address this gap, we investigated the *in vivo* effects of ET on breast cancer development using a mouse model. Specifically, we examined whether ET influences tumor aggressiveness and alters the expression of inflammatory markers in both the tumor tissues and spleen. By elucidating these interactions, our research aims to contribute to a deeper understanding of the interplay between immune tolerance and breast cancer.

## 2 Materials and methods

### 2.1 Experimental animals

**Figure 1:**
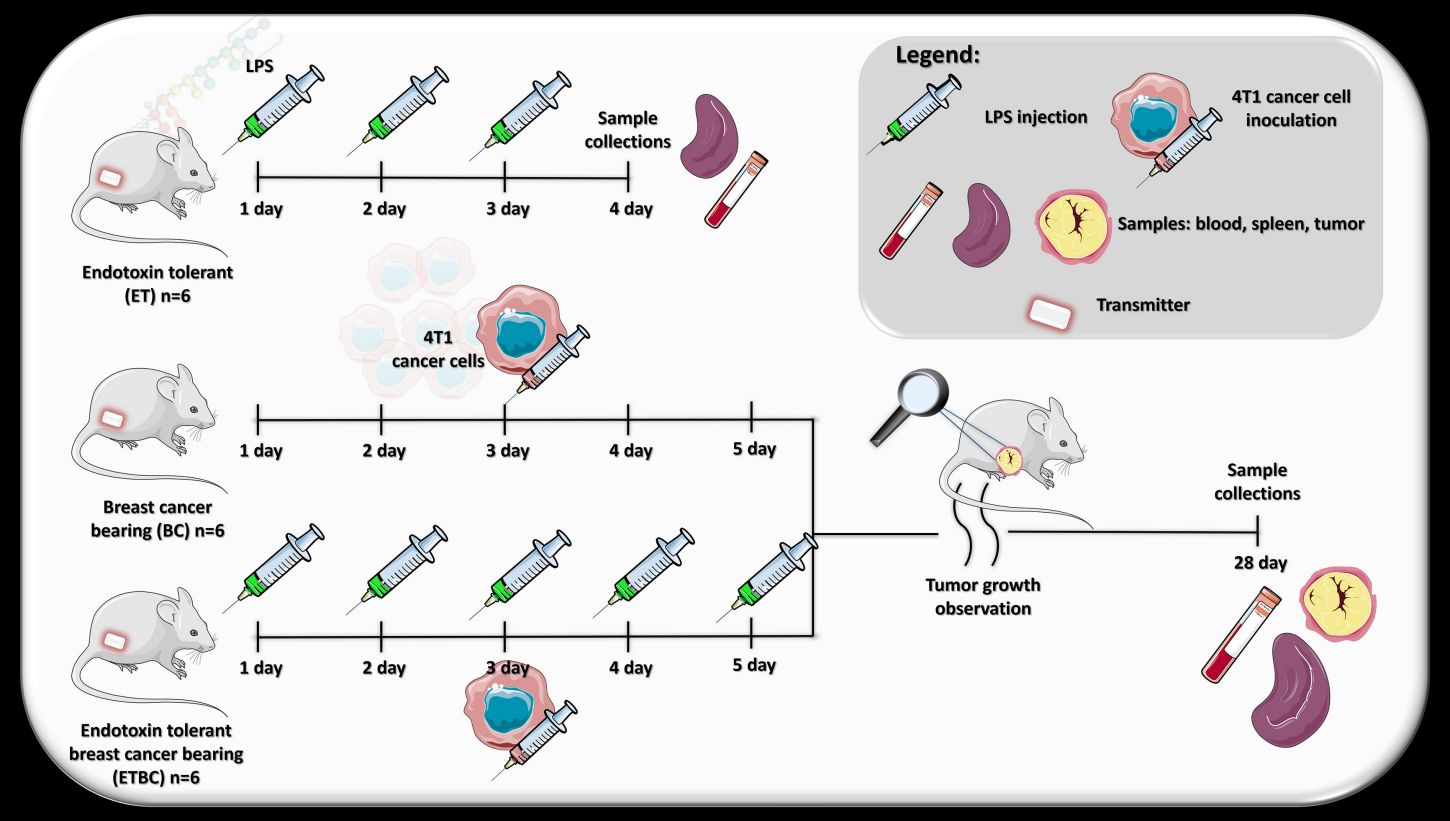
Scheme of experimental procedures conducted on the following groups of animals: endotoxin tolerant mice (ET), breast cancer-bearing mice (BC), and endotoxin tolerant breast cancer-bearing mice (ETBC).

This study adhered to the Animal Research: Reporting of *In Vivo* Experiments (ARRIVE) guidelines for the reporting of experimental results. Female BALB/c mice (6–8 weeks old) were purchased from the Mossakowski Medical Research Centre of the Polish Academy of Sciences (Warsaw, Poland) and allowed to acclimatize for 14 days before experimentation. Animals were housed individually in polycarbonate cages within a controlled environment. The room was maintained at a constant relative humidity of 50 ± 10% and a temperature of 24 ± 1 °C, with a 12-hour light-dark cycle, where lights were turned on at 7:00 a.m. Food and water were provided *ad libitum*. All procedures were approved by the Local Bioethical Committee for Animal Care in Bydgoszcz, Poland (permission no. LKE 50/2022). This research was conducted on four groups of mice: untreated (NT), endotoxin-tolerant (ET), breast cancer-bearing (BC), and endotoxin tolerant breast cancer-bearing (ETBC). **Figure 1** presents a diagram illustrates the procedure conducted for each group of animals.

### 2.2 *In vivo* procedure

#### 2.2.1 Temperature and motor activity measurement

The body temperature (T_b_) of the mice was monitored using temperature-sensitive miniature biotelemeters (PhysioTels model TA10TA-F40; Data Sciences International, St. Paul, MN, USA) implanted intra-abdominally in a sterile environment. Before transplantation, mice were anesthetized via intramuscular injection of a ketamine (80 mg/kg, Biowet, Puławy, Poland) and xylazine (10 mg/kg, ScanVet, Gniezno, Poland) mixture. A small area was then shaved and sterilized, after which an incision was made through the abdominal skin and muscle layers. Temperature-sensitive biotelemetry devices were inserted into the peritoneal cavity and the abdominal muscles and skin were sutured separately. All surgical procedures were completed at least 10 days before the start of the experiment.

The motor activity of the mice was monitored by tracking changes in the position of an implanted temperature-sensitive transmitter relative to a receiver board. These positional shifts resulted in variations in signal strength, which were detected by the external receiver antenna and recorded as “pulses” or “counts” of activity.

### 2.3 Inducing endotoxin tolerance in mice

Lipopolysaccharide (LPS) from *Escherichia coli* (0111:B4, Merck, Darmstadt, Germany) was prepared by dissolving LPS in sterile 0.9% sodium chloride. Prior to injection, the stock solution of LPS (2 mg/mL) was warmed to 37 °C, then diluted in warm sterile saline to the desired concentration. To induce ET, mice received daily intraperitoneal (i.p.) injections of LPS (50 μg/kg) four consecutive doses (**Figure 1**). ET development was assessed by measuring T_b_ of the mice using biotelemetry.

### 2.4 Inducing tumor and tissue collection

On the day of the third LPS injection, mice were inoculated subcutaneously (s.c.) with 2.5 × 10^4^ 4T1 breast cancer cells on the right flank (**Figure 1**). Before the experiments, 4T1 cells (American Type Culture Collection, Manassas, VA, USA) were cultured in high-glucose Dulbecco’s Modified Eagle’s Medium (DMEM) supplemented with 10% fetal bovine serum (FBS), 100 μg/mL streptomycin, and 100 IU/mL penicillin (all from Merck, Darmstadt, Germany). The cells were maintained at 37 °C in a humidified 5% CO_2_ atmosphere. The mice were monitored daily for tumor growth. After approximately four weeks, the mice were euthanized with an overdose of ketamine/xylazine (240 mg/kg and 30 mg/kg, respectively). Blood, tumor, and spleen samples were collected. Tumor and spleen samples were rinsed twice in sterile ice-cold PBS and immediately flash-frozen in liquid nitrogen (**Figure 1**). These tissue samples were stored at -80 °C for subsequent analysis.

### 2.5 Blood sample collection and blood morphology analysis

Blood samples were collected through cardiac puncture in EDTA-treated tubes from mice anesthetized with an overdose of ketamine/xylazine (240 mg/kg and 30 mg/kg, respectively). Total blood cell counts of leukocytes, lymphocytes, monocytes, and granulocytes were evaluated using the Auto Hematology Analyzer BC-2800Vet (Mindray, Shenzhen, China).

### 2.6 Measurement of the spleen size

Spleens were collected following euthanasia of the mice using an overdose of ketamine and xylazine mixture. Immediately after removal, the spleens were weighed using a highly sensitive scale to ensure precise measurement.

### 2.7 Histological assessment

Mouse mammary tumor tissues were assessed by pathologists. Tumor tissues were fixed in 10% neutral-buffered formalin for 24 h. Tissue samples were then routinely processed for histopathology and embedded in paraffin wax (FFPE). Paraffin sections of 5 μm thickness were stained for histology with Mayer’s hematoxylin and eosin (H&E). All images were captured using a Nikon Eclipse E800 microscope.

### 2.8 RNA extraction and RT-qPCR

Total RNA extraction from spleen and tumor tissues was performed by lysing them in PureZOL™ RNA Isolation Reagent with mechanical disruption, according to the manufacturer’s protocol. Then, cDNA was obtained using 1 μg of total RNA and the iScript™ cDNA Synthesis Kit, following the manufacturer’s protocol. RT-qPCR was performed in a final volume of 10 μL using SsoAdvanced Universal SYBR^®^ Green Supermix and PrimePCR™ SYBR^®^ Green Assays. **Table 1** lists the unique assay IDs of the immune-related gene primers. The test samples were analyzed in triplicate using a CFX Connect Real-Time PCR Detection System. The specificity was verified through melt curve analysis. Standard curves were generated for both target and reference genes (actin). Calibrator-normalized quantification was performed using the CFX Manager Software 3.1. Each reaction was performed at least twice. All reagents and software used to analyze cytokine expression were purchased from Bio-Rad (Hercules, CA, USA).

**Table 1:** The list of primers used for RT-qPCR.

Gene Name	Protein name	Unique assay ID
actin	ACTB	qMmuCED0027505
interleukin 6	IL-6	qMmuCED0045760
interleukin 1β	IL-1β	qMmuCED0045755
cyclooxygenase 2	COX-2	qMmuCED0047314
vascular endothelial growth factor	VEGF	qMmuCED0047509
interferon γ	IFN-γ	qMmuCID0006268
nitric oxide synthase 2 (NOS2)	inducible nitric oxide synthase (iNOS)	qMmuCID0023087
interleukin 10	IL-10	qMmuCED0044967
signal transducer and activator of transcription 6	STAT6	qMmuCID0006404
Colony stimulating factor 1	CSF-1	qMmuCID0019725

### 2.9 Survival analysis

The survival rates of the mice in the BC and ETBC groups were compared. The Kaplan-Meier plot was used to assess the differences, and statistical analysis between these groups was performed using the log-rank (Mantel-Cox) test and the Gehan-Breslow-Wilcoxon test. This analysis was performed using data obtained from an independent experimental set.

### 2.10 Tumor growth rate analysis

Tumor samples obtained from mice with BC and ETBC were measured using Vernier calipers. The tumor volume was calculated using the following formula:


$$V=\frac{\pi }{6}*L*W$$

where L represents the average tumor length and W represents the average width of the tumors. A comparison analysis between the BC and ETBC mice groups was conducted using nonlinear regression analysis.

### 2.11 Statistical analysis

The relative gene expression levels were evaluated and compared using the 2^−ΔΔCt^ method. Multiple group comparisons were performed using one-way analysis of variance (ANOVA), followed by the Sidak test. An unpaired t-test was used to evaluate gene expression between the two groups. For the evaluation of tumor growth rate, nonlinear regression with an exponential growth model was used. Tumor volume was calculated as (π/6) × width × length. Measurements were initiated once tumors were measurable in all mice, and follow-up measurements were taken after a few days to capture clear differences in growth between groups. The number of time points was limited to capture meaningful differences in tumor growth. GraphPad Prism version 8 software (GraphPad Software Inc., La Jolla, CA, USA) was used for calculations, analysis, and visualization of results. Statistical significance was set at p < 0.05.

## 3 Results

### 3.1 Repeated LPS injections at a constant dose result in a progressive and sustained reduction in fever and locomotor activity, indicating the development of endotoxin tolerance

**Figure 2:**
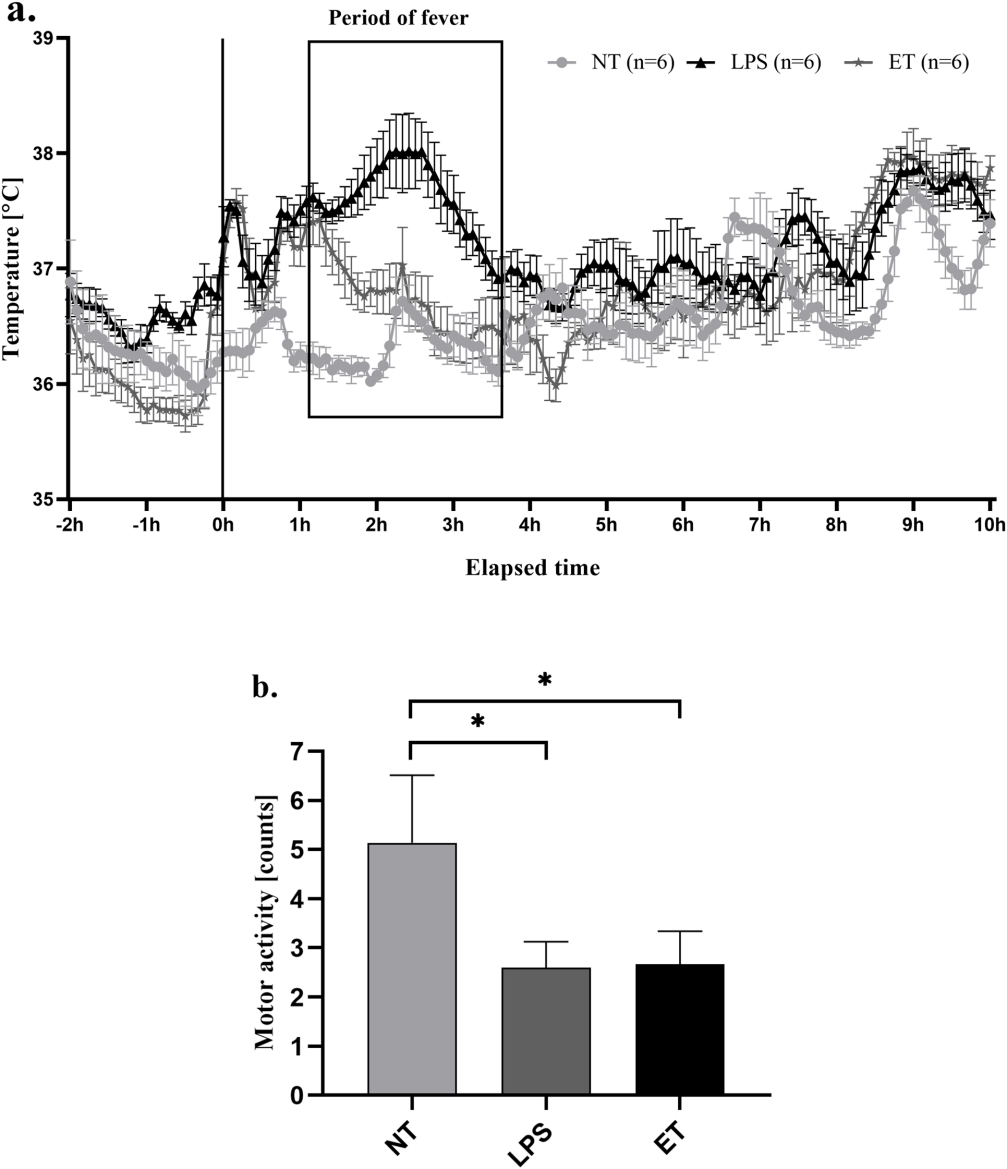
Body temperature and motor activity over a 12-hour period in mice under three different experimental conditions: non-treated (control) mice (NT); mice injected with a single dose of LPS (50 µg/kg, i.p.) to induce fever (LPS); endotoxin tolerant mice (ET). Representative time-course graph depicting core body temperature changes over a 12-hour period across the three groups **(A)**. Motor activity recorded during the same 12-hour period, demonstrating differences in activity levels between groups **(B)**. The asterisks indicate the significant difference between the groups indicated (*p < 0.05). Each group consisted of n = 6 mice. Data has been analyzed using one-way analysis of variance (ANOVA), followed by the Sidak test and these data are presented as mean ± SEM.

Mice are nocturnal animals with low daytime and high nighttime T_b_. Using four consecutive doses of LPS, we observed a progressive change in response to LPS. The first injection of LPS induced fever, which started within 1 h of LPS injection. The occasional transient increase in the T_b_ of mice at 9 a.m. (injection time) was mainly caused by stress related to the injection and handling of the mice. Fever onset was achieved and maintained for approximately one hour, with the highest body temperature reaching 38.02 ± 0.33 °C, compared to 36.72 ± 0.21 °C in NT mice (p < 0.001). This was followed by a fever duration of roughly 1.5 hours before the gradual return of body temperature to baseline levels. Repeated daily injections of LPS over several days resulted in progressive attenuation of the febrile response, culminating in the establishment of the ET model in mice. By the fourth dose of LPS, the maximum temperature in the treated group was 37.03 ± 0.33 °C, compared to 38.02 ± 0.33 °C following the first dose of LPS (p < 0.001) (**Figure 2A**).

Another important aspect of ET is sickness behavior, such as a decrease in motor activity. Indeed, a significant reduction in locomotor activity was observed in mice exhibiting ET compared to that in NT animals. The average motor activity value recorded was 2.66 ± 0.67 counts in ET mice and 2.6 ± 0.52 counts in LPS mice when compared to 5.14 ± 1.37 counts in NT mice after the LPS injection (p < 0.05) (**Figure 2B**). The reduction in activity, typically associated with fever, was evident in LPS-treated mice and persisted throughout the development of ET.

The absence of a systemic febrile response, a hallmark of ET, indicates successful immune reprogramming, thereby validating the robustness of this approach. In subsequent experiments, breast cancer was induced in ET mice, and its progression was compared to that in non-endotoxin-tolerant mice.

### 3.2 Microscopic evaluation of 4T1-induced mammary tumors in mice

**Figure 3:**
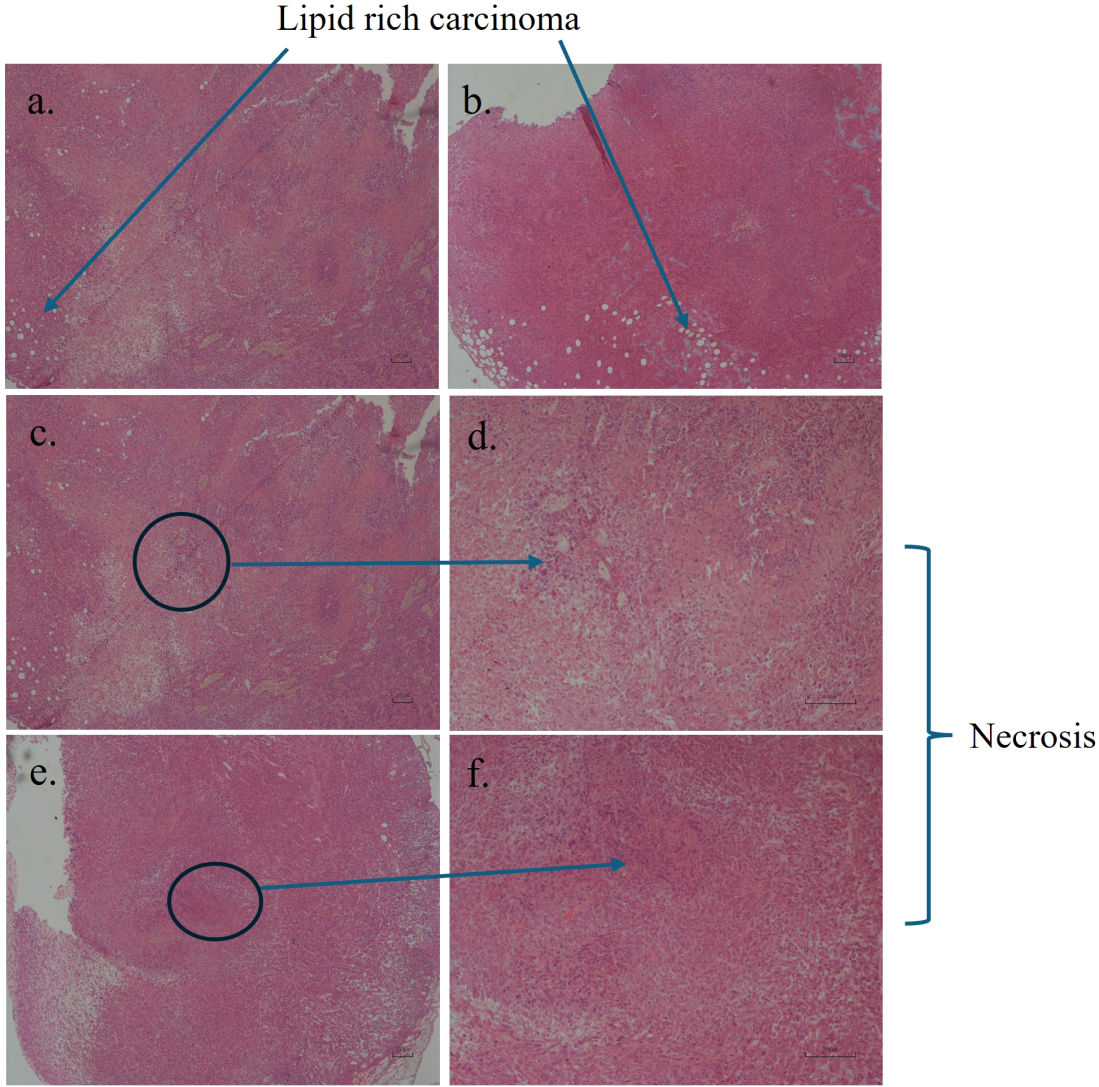
Representative images of breast tumor tissues. Lipid-rich carcinoma was observed in both the breast cancer-bearing mice [BC; **(A)**] and endotoxin tolerant breast cancer-bearing mice [ETBC; **(B)**]. Necrotic areas were identified in both BC **(C, D)** and ETBC **(E, F)** animals. Necrotic tissue images were captured at 4× and 10× magnification, respectively, for both groups. Histological analysis was performed on six mice per group.

Six days after 4T1 cell injection, palpable breast tumors were observed in mice. These tumors were allowed to develop in the mice until the study’s conclusion on day 28; subsequent histological analysis showed solid sheets of proliferating epithelial cells, polygonal to oval-shaped, with poorly demarcated margins and scant cytoplasm. Nuclei are vesicular, with coarse chromatin, and a single central basophilic nucleus. There is a very fine fibrovascular connective tissue that rarely subdivides the neoplasm into lobules. Tubular differentiation was not observed, and the neoplastic cells were pleomorphic with numerous mitoses. The majority of the ETBC mice were consistent with solid carcinoma. Both the ETBC and BC groups showed numerous cells with large and small vacuoles in the cytoplasm and nucleus, which are often located at the periphery of the cell. These results are consistent with those of lipid-rich carcinoma (**Figures 3A, B**). Necrotic areas were present in all samples in different amounts and were surrounded by a small number of macrophages and very few neutrophils (**Figures 3C–F**).

### 3.3 Endotoxin tolerance influences the survival and tumor growth of the mice

**Figure 4:**
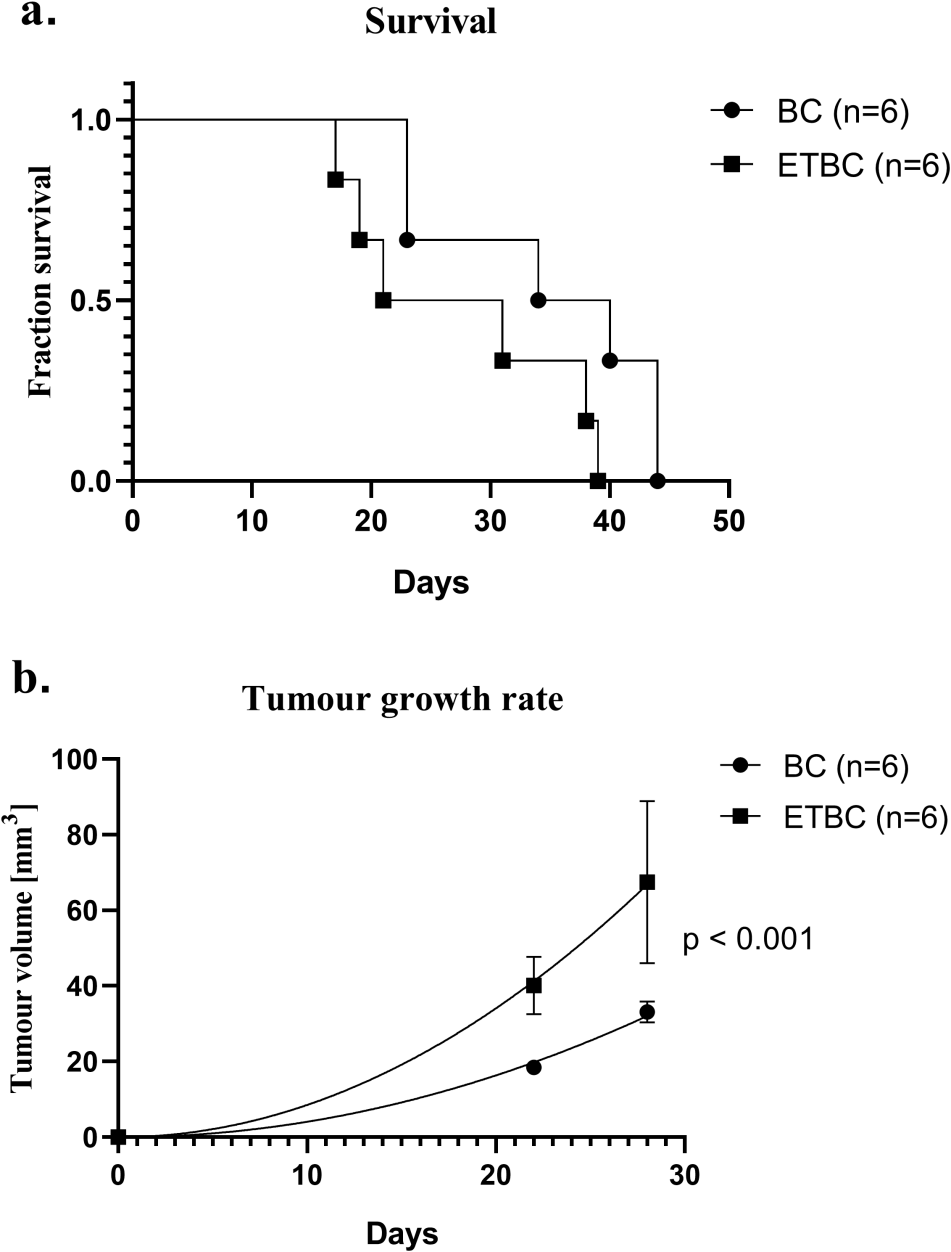
Kaplan–Meier mice survival curve **(A)** and tumor volume **(B)** in cancer-bearing mice (BC) and mice with endotoxin tolerance and cancer (ETBC). The experiments were performed on 6 individuals in each group (n=6). The survival analysis was performed using the log-rank (Mantel-Cox) test and the Gehan-Breslow-Wilcoxon test, while the comparison of the tumor volume was performed using nonlinear regression with an exponential growth model. The non-linear regression curves revealed a significant difference between groups (p < 0.001).

Having established a stable model of ET manifested by the absence of fever, we studied its role in modulating tumor progression and overall survival outcomes in an experimental model of breast cancer. We observed a difference in the survival capacity of ETBC mice compared to that of BC group animals (**Figure 4A**). Although the difference was not statistically significant (p = 0.0710), mice with ETBC reached an advanced stage of the experiment more rapidly. Notably, after reaching the advanced stage of cancer, their condition deteriorated at a faster rate than that in BC mice (data not shown). None of these mice allowed for the continuation of observation beyond 40 days from the induction of breast cancer. In contrast, BC mice exhibited slower disease progression, and their condition allowed observation for up to 45 days (**Figure 4A**).

Mice in the ETBC group exhibited a more rapid increase in tumor volume, indicating that ET may enhance tumor progression. This accelerated tumor growth was accompanied by an earlier onset of advanced disease stages, which required humane termination of the experiment. In contrast, tumor progression in the BC group was slower, with some mice maintaining stable conditions for a longer duration. The following data were derived from three time points recorded during our observational study, with the final measurement being day 28. The tumor volume of the BC group on day 22 was observed to be 18.4 ± 1.8 mm^3^ and it reached 33 ± 2.8 mm³ by day 28. On the other hand, the mean tumor size in the ETBC group was 40.1 ± 7.5 mm³ on day 22, which then reached 67.4 ± 21.4 mm³ by day 28. The comparison of nonlinear regression curves revealed a significant difference between the groups (p < 0.001) (**Figure 4B**). These findings suggest that ET can exacerbate tumor growth dynamics, potentially through alterations in the immune response.

### 3.4 Impaired leukocyte recruitment in endotoxin tolerant mice with cancer

**Figure 5:**
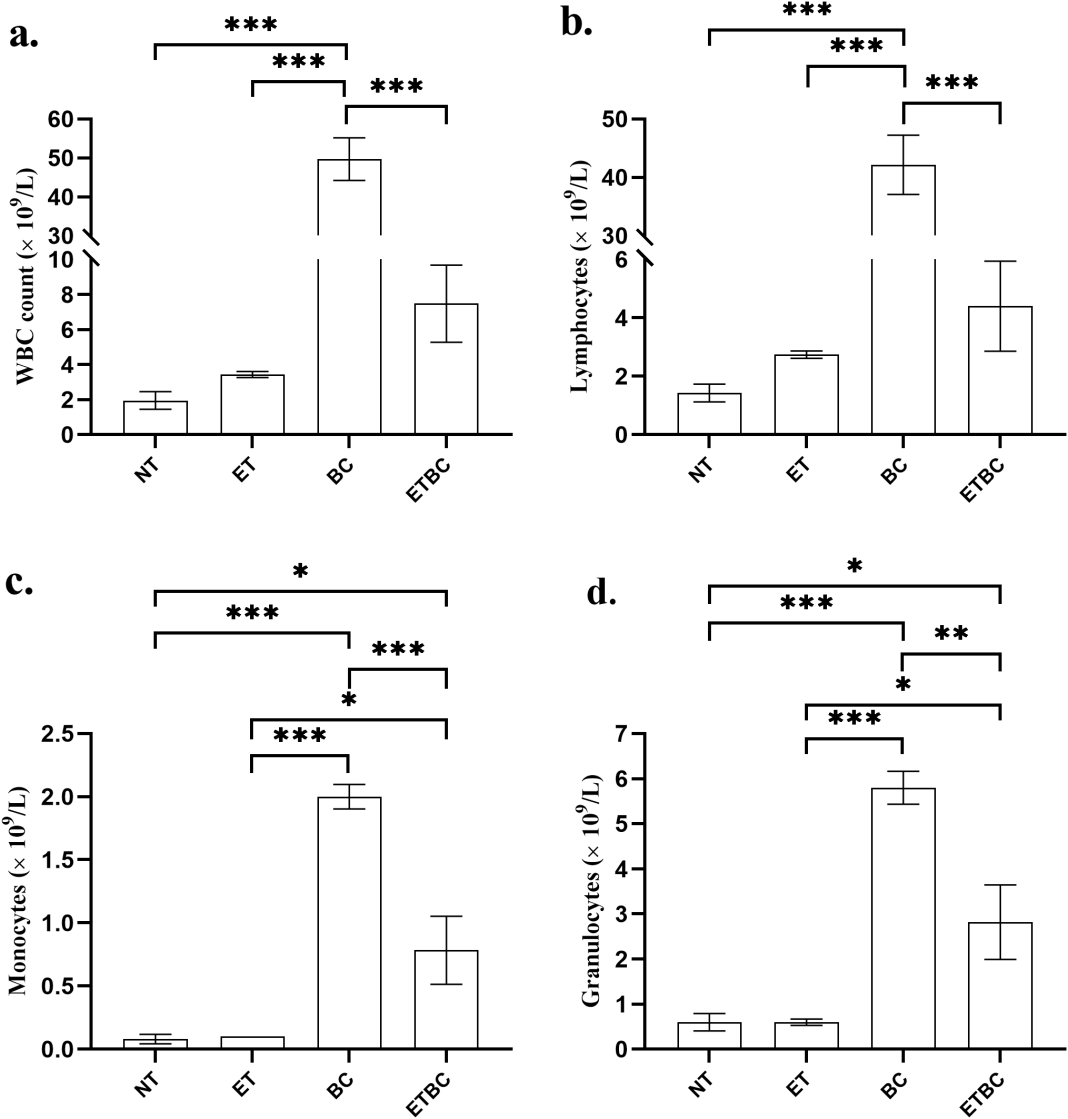
Effect of endotoxin tolerance (ET) and breast cancer on the total count of white blood cells **(A)**, lymphocytes **(B)**, monocytes **(C)** and granulocytes **(D)**. Figure represents the mean ± SEM of the leukocytes, measured in mice. The one-way analysis of variance (ANOVA), followed by the Sidak test was used for this analysis. The asterisks (*) indicate the significant difference between the groups indicated (***p < 0.001, **p < 0.01, *p < 0.05). Control animals (NT; n=5), endotoxin tolerant mice (ET; n=5), cancer-bearing mice (BC; n=6) and cancer-bearing mice with ET (ETBC; n=6).

To better understand how ET influences immune system function, white blood cell (WBC) analysis was conducted in four groups of mice. We observed that the general leukocyte counts in NT and ET mice were almost similar (p > 0.99), whereas a significant increase was observed in the BC and ETBC groups (p < 0.001) (**Figure 5A**). A detailed analysis of individual leukocyte populations revealed that this increase was observed across all measured leukocyte populations, including lymphocytes, monocytes, and granulocytes (**Figures 5B–D**, respectively). Interestingly, leukocyte counts in cancer-bearing mice were significantly lower in ETBC animals than in BC mice (p < 0.001).

### 3.5 Tumor-induced splenomegaly is not observed in endotoxin tolerant mice

**Figure 6:**
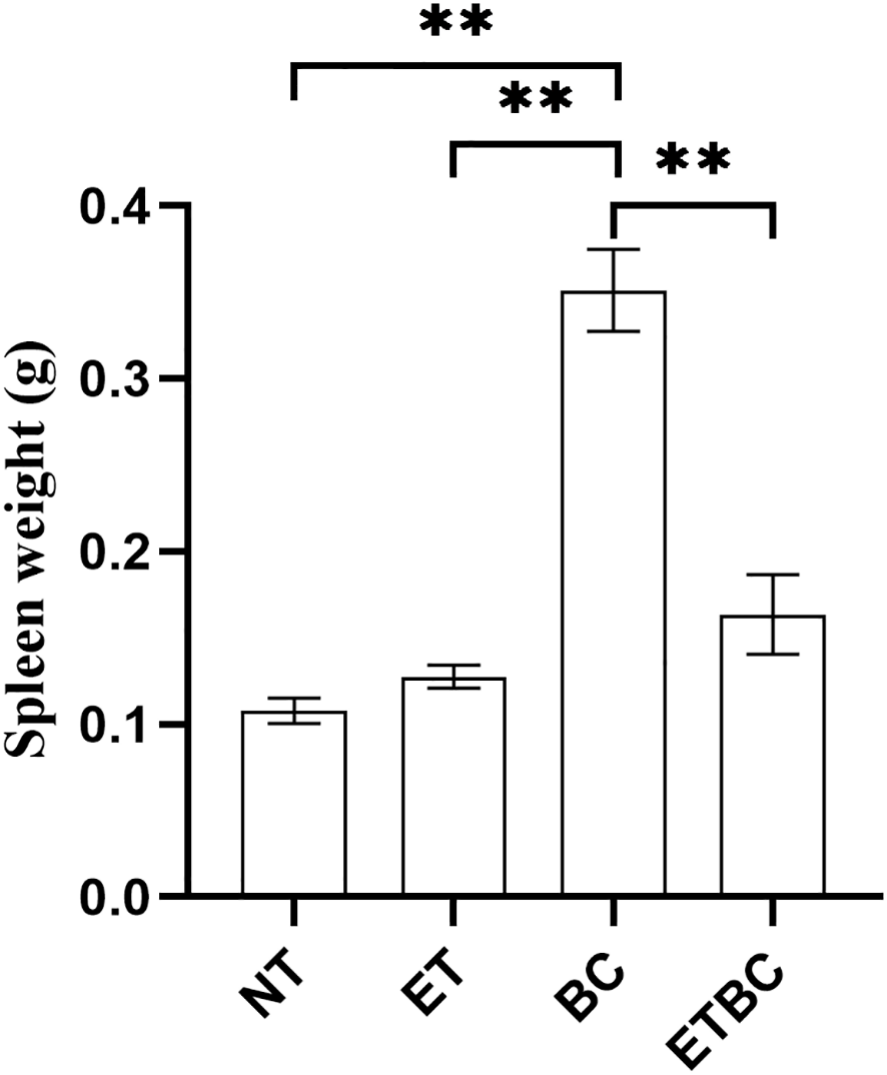
Effect of endotoxin tolerance (ET) and breast cancer on the spleen weight. Figure represents the mean ± SEM of the spleen weight in each group. The asterisks indicate the significant difference between the groups indicated (**p < 0.01). The one-way analysis of variance (ANOVA), followed by the Sidak test was used for this analysis. Control animals (NT; n=5), endotoxin tolerant mice (ET; n=5), cancer-bearing mice (BC; n=6) and cancer-bearing mice with ET (ETBC; n=6).

The spleen is a major reservoir of immune cells and plays a crucial role in the regulation of immune response. We observed distinct differences in spleen size between groups, which further underscored the impact of ET on immune function. Spleen samples collected from BC mice were noticeably larger in size, indicating splenomegaly. Notably, spleens from ETBC animals weighed significantly less than those from the BC group (p < 0.01). Additionally, spleen weight in BC mice was significantly higher than that in the NT and ET groups (p < 0.01) (**Figure 6**).

### 3.6 Endotoxin tolerance and breast cancer modulate immune-related genes in spleen

**Figure 7:**
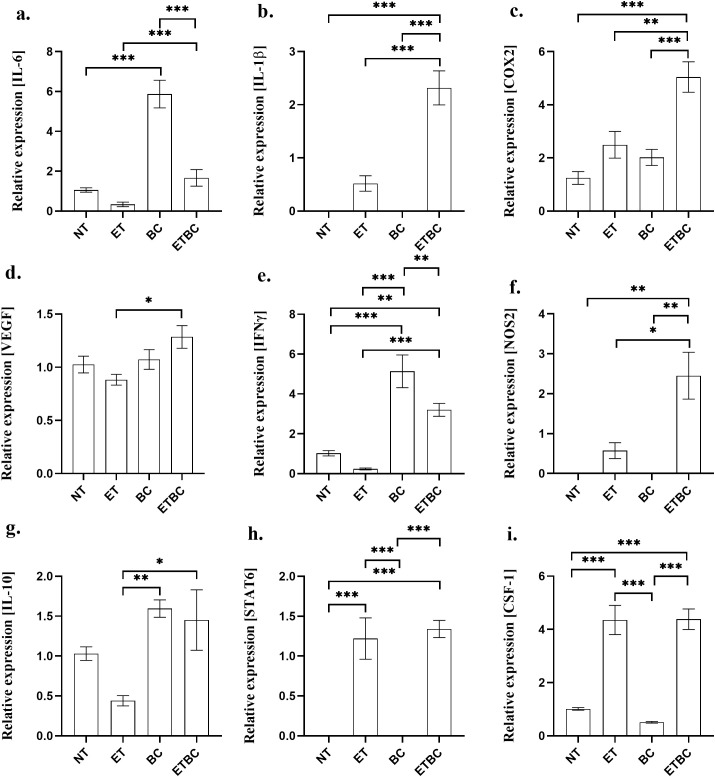
Expression of immune-related genes: IL-6 **(A)**, IL-1β **(B)**, COX-2 **(C)**, VEGF **(D)**, IFN-γ **(E)**, NOS2 **(F)**, IL-10 **(G)**, STAT6 **(H)**, and CSF-1 **(I)** in spleen from the following groups of animals: control mice (NT; n=10), endotoxin tolerant mice (ET; n=10), cancer-bearing mice (BC; n=14) and cancer-bearing mice with ET (ETBC; n=14). The asterisks indicate the significant difference between the groups indicated (*p < 0.05, **p < 0.01 and ***p < 0.001). Relative gene expression levels were calculated using the 2^−ΔΔCt^ method. Statistical analysis was performed using one-way analysis of variance (ANOVA), followed by Sidak’s multiple comparisons test.

The blood morphology results showed some abnormalities i.e., the elevated leukocyte count in both BC and ETBC groups (**Figure 5**); therefore, gene expression analysis of immune-related genes in the spleens was carried out. We examined the expression of proinflammatory genes, including IL-6 (**Figure 7A**), IL-1β (**Figure 7B**), COX-2 (**Figure 7C**), VEGF (**Figure 7D**), IFN-γ (**Figure 7E**), and NOS2 (**Figure 7F**). Additionally, we investigated the expression of genes associated with immune regulation or inflammation: IL-10 (**Figure 7G**), STAT6 (**Figure 7H**), and CSF-1 (**Figure 7I**). The results showed a significant decrease in the expression of IL-6 and IFN-γ in mice with ETBC compared primarily to that in BC animals (p < 0.001 and p < 0.01, respectively). However, compared to the ET group, the expression of IL-6 and IFN-γ in the ETBC group remained significantly increased (p < 0.001).

Moreover, an increase in the expression of NOS2 (p < 0.001), IL-1β (p < 0.001), STAT6 (p < 0.001), CSF1 (p < 0.001), and COX-2 (p < 0.001) was observed in the ETBC group, notably when compared to the BC group. The expression of NOS2, IL-1β, and COX-2 also remained significantly higher in the ETBC group than in the NT (p < 0.001) and ET groups (p < 0.05, p < 0.001, and p < 0.01, respectively). Additionally, IL-10 expression, though insignificant, has been observed to be higher in ETBC group when compared to NT group (p > 0.6) and significantly higher when compared to ET group (p < 0.01). Similarly, STAT6 and CSF-1 expression levels were significantly higher in the ETBC group than in the NT group (p < 0.001) (**Figure 7**).

### 3.7 Endotoxin tolerance modulates immune-related genes in tumor tissues

**Figure 8:**
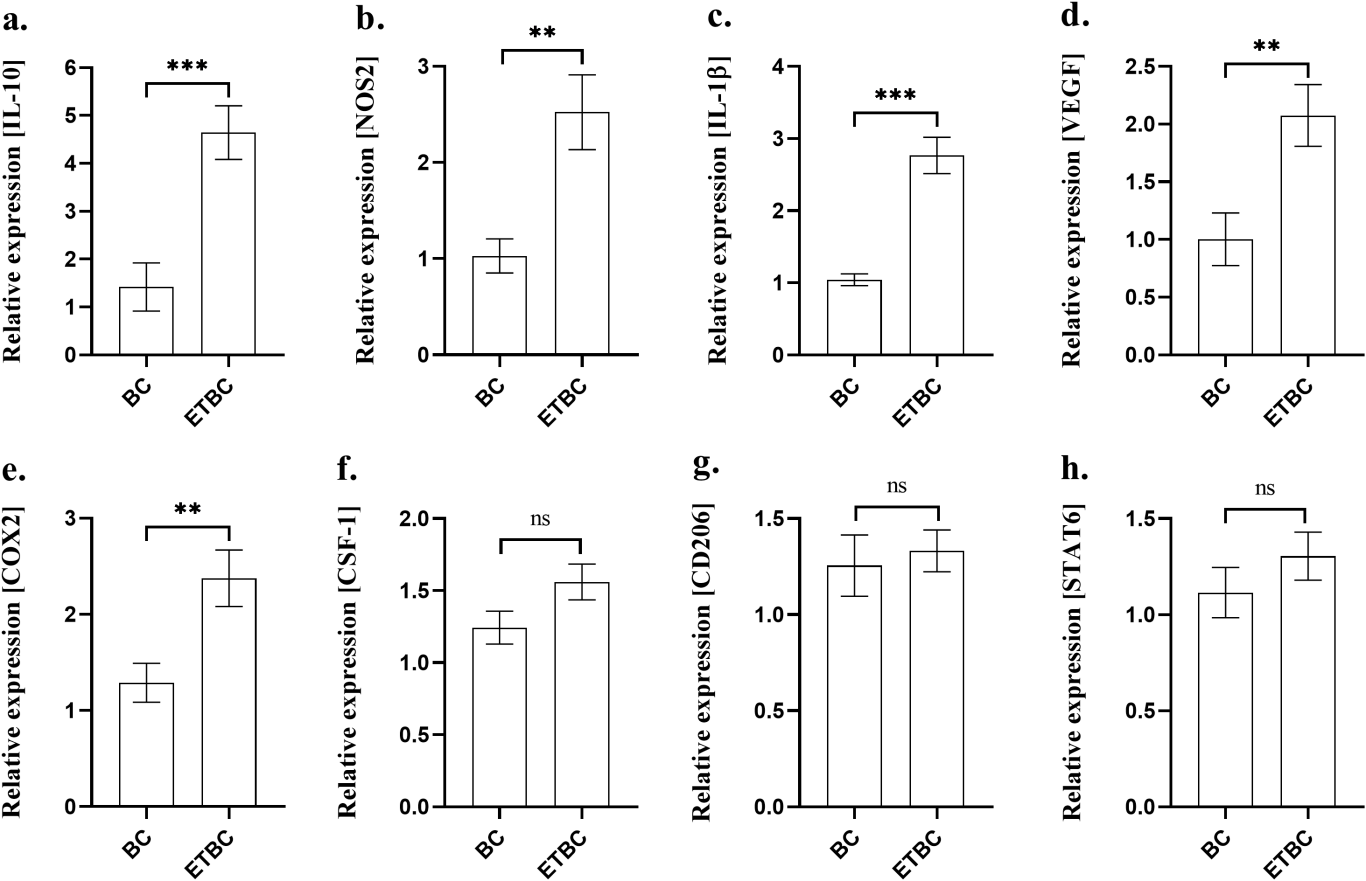
Expression of immune-related genes: IL-10 **(A)**, NOS2 **(B)**, IL-1β **(C)**, VEGF **(D)**, COX-2 **(E)**, CSF-1 **(F)**, CD206 **(G)** and STAT6 **(H)** in tumor tissues from the following groups of animals: cancer-bearing mice (BC; n=14) and cancer-bearing mice with ET (ETBC; n=14). Relative gene expression levels were calculated using the 2^−ΔΔCt^ method, and differences between the two groups were evaluated using an unpaired t-test. The asterisks indicate the significant difference between the groups indicated (**p < 0.01, ***p < 0.001; ns – not significant).

Finally, we wanted to determine whether ET affected the expression of immune-related genes within the tumor itself. We analyzed the expression of IL-10, NOS2, IL-1β, VEGF, COX-2, CSF-1, CD206, and STAT6 (**Figures 8A–H**, respectively) in tumor tissues from BC and ETBC mice. We observed a significant increase in the expression of IL-10, NOS2, IL-1β, VEGF, and COX-2 in ETBC mice when compared to BC mice (p < 0.01, p < 0.001). Although not statistically significant, the expression of CSF-1, CD206, and STAT6 was higher in the ETBC group than in the BC group (**Figure 8**).

## 4 Discussion

Endotoxin tolerance (ET) is a phenomenon in which the immune system becomes less responsive to endotoxins after repeated exposures, leading to alterations in immune function. Using a mouse model of ET, we confirmed the absence of fever following administration of a pyrogenic dose of endotoxin. This is a hallmark feature of ET, indicating immune system reprogramming (19). The inability to generate fever is particularly noteworthy as it may indicate underlying immune alterations. These changes could have significant implications for infection management and, less commonly, cancer development. Little attention has been paid to atypical infection courses in oncology patients, which are often marked by rare or absent febrile episodes. In this study, we tested the hypothesis that endotoxin tolerance may create an immunosuppressive environment that promotes breast cancer progression. For our study, we used a model in which tumor cells are introduced during the early phase of ET, as this setup reflects a clinically relevant scenario in which chronic or recurrent endotoxin exposure (e.g., during persistent inflammation or dysbiosis) may coincide with early tumor development. Although the anti-tumor effects of bacterial components have been reported in several studies (20–24), we did not observe tumor remission in our model, likely due to the presence of established endotoxin tolerance, which attenuates the host’s responsiveness to bacterial stimuli.

As expected, our endotoxin tolerant mice did not develop a fever in response to a pyrogenic dose of LPS. Moreover, reduced locomotor activity was observed after each LPS injection even after ET development. These findings indicate that ET selectively dampens the febrile response, whereas sickness behavior, such as reduced locomotor activity, persists. This suggests that distinct mechanisms may underlie different components of the systemic response to LPS.

In the context of cancer, we observed that ETBC mice showed more rapid tumor progression and earlier disease onset than the BC group. The condition of these mice deteriorated at a faster rate after reaching advanced stages of cancer, suggesting that ET creates an environment that exacerbates tumor growth. This finding supports previous studies that indicated that immune reprogramming as a result of ET might undermine the immune system’s capacity to fight cancer, thereby promoting tumor growth (10, 25–27).

In our study, we analyzed blood morphology to provide a direct measure of the changes in immune cell populations and overall immune function. By analyzing parameters such as leukocyte count and distribution of different immune cell types, we were able to identify potential alterations in immune responses caused by ET. We found a significant difference in leukocyte counts between the ETBC and BC mice. In the BC group, there was a notable increase in leukocyte populations, including lymphocytes, monocytes, and granulocytes, which are typically recruited as part of the immune response to tumor growth. The increase in leukocytes in these mice is consistent with tumor-associated inflammation, which recruits immune cells to fight the tumor (28–32). By contrast, ET suppressed leukocyte recruitment in the ETBC group. Specifically, the leukocyte count was significantly lower in ETBC mice than in BC mice, suggesting that ET may impair immune cell mobilization, resulting in an ineffective immune response to cancer (33–36).

Additionally, we investigated the role of the spleen, a key organ in immune cell storage and activation. The spleen plays a critical role in regulating immune responses, particularly during inflammation and infection (37–40). We observed that in BC mice, spleen weight was significantly increased, reflecting enhanced immune cell recruitment as part of the inflammatory response to tumor growth. However, in the ETBC group, spleen weight was significantly reduced despite the presence of cancer. This suggests that ET might impair immune cell mobilization or alter the immune function of the spleen, which could suppress the overall immune response to the tumor and contribute to more rapid tumor progression. The reduced spleen size and lower leukocyte count in ETBC mice further supports the notion that ET hampers immune system effectiveness in combating cancer (10, 19).

To evaluate changes in gene expression related to cancer and inflammatory response, we analyzed RNA isolated from the spleen. Our findings revealed a significant reduction in the expression of IL-6 and IFN-γ in the ETBC group compared to the BC group, suggesting that ET suppresses pro-inflammatory cytokines that typically contribute to immune activation. However, these levels remained significantly higher in the ETBC group than in the ET group, indicating that cancer may still modulate the immune response, even in the presence of ET (41, 42). Interestingly, the ETBC group exhibited elevated expression of NOS2, IL-1β, and COX-2, which are associated with inflammation and immune regulation. These cytokines are involved in both promoting and resolving inflammation, suggesting that ET may lead to an altered immune environment, potentially contributing to immune evasion of the tumor (43–45). This complex immune modulation highlights the interaction between ET and cancer, where ET does not entirely suppress the inflammatory response, but may instead redirect it in a way that enhances tumor progression.

Analysis of immune-related gene expression in tumor tissues also revealed that ET influences the tumor microenvironment by increasing the expression of IL-10, NOS2, IL-1β, VEGF, and COX-2 in the ETBC group compared with that in the BC group. These genes play a crucial role in tumor progression, inflammation, and immune regulation, and their elevated expression in ETBC mice suggests that ET may enhance certain inflammatory pathways within the tumor (46–48). The expression of CSF-1, CD206, and STAT6 remained higher in the ETBC group, although these differences were not statistically significant, indicating a potential trend toward immune regulation, which requires further investigation (49–51). Overall, the increased expression of IL-1β, NOS2, COX-2, and VEGF in ETBC tissues likely reflects persistent reprogramming of the inflammatory response and the tumor microenvironment toward a pro-tumorigenic state. At the same time, reduced peripheral leukocyte counts and decreased spleen size indicate suppression of systemic immunity. Together, these findings suggest that prior ET promotes later local pro-inflammatory and pro-angiogenic signaling within the tumor while simultaneously impairing immune surveillance, thereby facilitating tumor growth and progression.

While our study highlights ET-mediated immunosuppression as a major contributor to accelerated tumor growth in ETBC mice, we acknowledge that other mechanisms may also play a role. To address this, future studies are needed employing adoptive transfer of immune cells, selective macrophage depletion, and TLR4 antagonism to dissect the specific contributions of ET versus alternative pathways. Such mechanistic investigations will clarify whether the tumor-promoting effects observed are solely dependent on ET-induced macrophage reprogramming or involve additional immune and tumor-intrinsic factors.

## 5 Conclusion

In summary, our study demonstrated that ET, characterized by the absence of fever, may lead to immune dysregulation, impairing the body’s ability to mount an effective defense against tumors. This immune reprogramming may contribute to enhanced tumor progression, as evidenced by more rapid growth and earlier disease onset in endotoxin-tolerant mice. Furthermore, ET alters immune responses at both systemic and local levels, influencing leukocyte counts, spleen size, and gene expression profiles related to inflammation and immune regulation. These findings highlight the complex interactions between ET and cancer, suggesting that ET may create an immune environment that favors tumor growth.

Moreover, our results suggest a possible explanation for why oncology patients often have fever-free medical histories (11, 12, 52). The absence of fever may reflect an underlying state of ET, leading to immune suppression and facilitation of tumor progression. Conversely, the occurrence of fever during cancer, which indicates overcoming tolerance, could play a crucial role in triggering immune activation and potentially driving tumor regression.

## Data Availability

The original contributions presented in the study are included in the article/supplementary material. Further inquiries can be directed to the corresponding authors.
